# Monte Carlo simulation of laser beams interaction with the human eye using Geant4

**DOI:** 10.1186/1475-925X-13-58

**Published:** 2014-05-07

**Authors:** Diogo Tendeiro, Gonçalo Lopes, Pedro Vieira, José Paulo Santos

**Affiliations:** 1 Centro de Física Atómica, CFA, Departamento de Física, Faculdade de Ciências e Tecnologia, FCT, Universidade Nova de Lisboa, 2829-516 Caparica, Portugal

**Keywords:** Retinal drusen, Computer simulation, Monte Carlo method, Geant4, Diagnostic techniques, Ophthalmological

## Abstract

**Background:**

Due to the unique characteristics of the eye, ophthalmologic diagnostic techniques often rely on the photons interaction with the retina to infer its internal structure. Although these techniques are widely used, the interpretation of the generated images is not always fully understood, as in scanning laser ophthalmoscopy dark field imaging. This limits the exploitation of its full potential as a diagnostic tool for deep abnormalities in the retina, as in the situation of drusen.

**Methods:**

With the aim of better understanding the retinal diagnostic images, we have carried out computer simulations of incident laser beams interacting with different structures of the human eye, including a retina with and without drusen. We have used the Geant4 simulation toolkit, applying the optical package of the electromagnetic (EM) physics working group, to simulate the physical processes of reflection, refraction, absorption, and scattering of low energy photons (2 eV) in biological tissues. For each simulation it was used a single beam of orange light, with a Gaussian profile, that travels through all optical elements of the eye. The reflected beam characteristics were analyzed by virtual detectors in different locations, which collected information about the number and position of photons. The geometry and optical properties of all components of the eye were considered according to the published data.

**Results:**

Simulation results put in evidence that the presence of drusen influences the profile of the reflected beams. It changes the mean free path of the photons, modifying its reflection pattern, which depends on the area illuminated by the incident beam. This result is also visible when the reflected beam is analyzed outside of the eye, when the profile has no longer a symmetrical Gaussian distribution. These results will support the retinal diagnostic images that will be obtained in a near future with a new developed ophthalmic apparatus.

**Conclusions:**

The shape analysis of the reflected beams in retinal laser scanning techniques could increase its potential as a diagnostic examination tool for the deeper structures of the retina.

## Background

Due to the unique characteristics of the eye, consisting of a transparent lens that allows direct retinal observations using photons in the visible range, ophthalmologic diagnostic techniques often rely on the photons interaction with the retina to infer its internal structure. Examples of those techniques are the conventional fundus imaging, optical coherence tomography (OCT) or scanning laser ophthalmoscopy (SLO). Despite the generalized use of these techniques, often the interpretation of the produced images is not fully understood. This happens, for example, with the images collected by the SLO configuration known as indirect mode, dark field or retro-mode [1]. Although the images generated by this imaging mode contain medical relevant information, a deeper understanding of the photons interaction with the different structures of the eye is needed. A particular example of this situation is the presence of drusen in the retina [2], whose detection is relevant for an earlier diagnosis of diseases as the age-related macular degeneration (AMD), which is a major cause of blindness [3]. In an earlier stage of development, these structures are very difficult to detect by conventional techniques but more evident in the retro-mode. The objective of this work is to develop the necessary simulation tools to study the mechanisms of the interaction of light with different eyeball structures, including the retina, in order to better understand the results of retinal laser scanning techniques and to find a practical analysis method to detect drusen in early stages of development. To accomplish this goal, it was used the Monte Carlo (MC) simulation technique [4].

The MC simulation is a powerful and flexible tool for simulating various physical phenomena, since it eliminates the need of analytical solutions associated with complex and difficult problems. Consequently, the MC approach is more flexible and suitable for analyzing a wider variety of radiation problems than analytic methods.

Many physics based MC codes, such as EGS [5], FLUKA [6], and MCNP [7], have been developed and applied for radiation research. More recently, Geant4 [8,9], a collection of C++ class libraries, originally developed for high-energy physics detector simulation, has found extensive use in the analysis of radiation and particle effects on low-medium-energy physics [10]. The history of Geant4 was documented by Agostinelli et al. [8]. The project’s initial goal was to develop a modern, object-oriented framework in which to build radiation-modeling tools. Recently, Geant4 has been recognized the ability to work with low energy beams, making this an appropriate tool to study the interaction of low energy light beams with biological samples. An example of this evolution is the Geant4-DNA project [11].

## Methods

### Model

The study of the passage of radiation in biological media is a growing area of research within the field of biomedical optics. Modeling the related processes in scattering media cannot be easily described by analytical models of radiation transport, and instead requires the use of stochastic Monte Carlo (MC) methods.

Actually, there are several publicly available MC codes that may be used to describe photon propagation in turbid media for biomedical applications [12-15]. However, these packages are not well suited to accurately consider all aspects of the generation and transport of photons. In this work, it is used the Geometry and Tracking (Geant4) software package (version 4.9.4) to simulate the passage of light through the human eye, which is an object-oriented toolkit for the simulation of particle propagation through matter [16]. It makes use of a large number of physics models to simulate radiation transport of various particles, with different energy values, through matter. This Geant4 package has been extensively validated, and has been utilized to describe transport in the areas of high-energy, nuclear, space, and medical physics, among others [17,18]. Recently, Glaser et al. [19] published a rigorous validation of the Geant4 for light transport in comparison to accepted standards within the biomedical optics community.

In the very low energy region, Geant4 supports two databases packages: the low energy package and the Penelope package. The low energy package includes the photoelectric effect, Compton scattering, Rayleigh scattering, gamma conversion, Bremsstrahlung, ionization and fluorescence of excitation of atoms [20].

In Geant4, the user can build a particular virtual simulation scene by defining the geometry and composition of the media, the particles and the physical processes. The kernel, considering the material properties and the selected physical processes, tracks all interactions of the primary and secondary particles throughout the virtual structures.

The virtual simulation scene of this work consists of a parallelepipedic world volume, filled with air, which has inside of it other volumes of different sizes and shapes. All these different volumes form together the virtual eyeball used to perform the simulations. The virtual eyeball was built using some of the classes of three-dimensional shapes provided by Geant4, specifically, *G4Sphere* and *G4Ellipsoid*. The dimensions of the different volumes were defined after a bibliographic survey of the real dimensions of the anatomical structures of the human eyeball [21].

Concerning the crystalline lens, which may present different shapes and axial dimensions during the process of visual accommodation (contraction or relaxation of the ciliary muscles) and varies among different individuals (genetic variability), it was assumed that has a format similar to a biconvex lens with the following average dimensions [22]:

• Equatorial diameter (ciliary muscles relaxed): 9.03 ± 0.30 mm

• Thickness (ciliary muscles relaxed): 3.69 ± 0.25 mm

Considering these dimensions, the crystalline lens was represented in Geant4 by a *G4Ellipsoid* with a diameter of 9 mm and a thickness of 3.7 mm. It is worth to mention that the slight variation in the lenses dimensions originate a change of the beams divergence that can be easily compensated by tuning the parameters of the diagnostic appliance.

**Figure 1:**
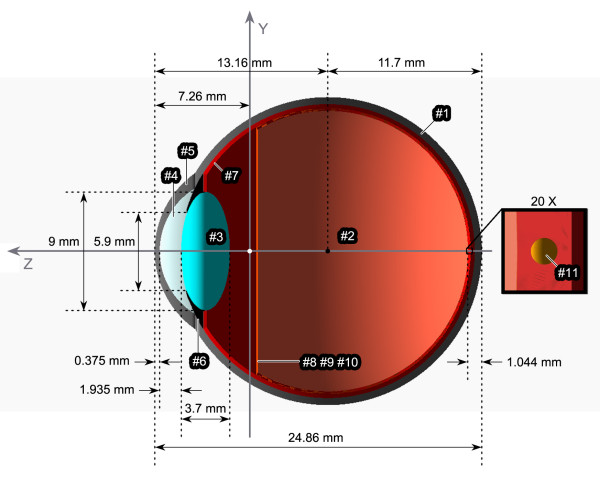
**Schematic drawing of the simulated virtual eyeball (sagittal section).** #1 - Sclera; #2 - Vitreous humor; #3 - Crystalline lens; #4 - Aqueous humor; #5 - Cornea; #6 - Iris; #7 - Choroid; #8 - Internal limiting membrane (ILM); #9 - Neurosensory retina (NR); #10 - Retinal pigment epithelium (RPE); #11 - Spherical druse (Olho_S1 program). The vertical red line (#8 #9 #10) indicates the ora serrata, that marks the transition from the non-photosensitive area of the retina to the multi-layered photosensitive region, i.e. the end of retinal layers.

Some volumes were positioned within other volumes giving rise to the different layers and cavities presented in the human eye. The retina was represented by three different layers [23]. In order to avoid overlapping of some volumes and to promote the full inclusion of them in their mother volumes, Boolean operations were used to build different and more complex shapes. We constructed a simple and functional model of the eyeball, corrected in terms of average anatomical characteristics, which is represented in Figure 1.

In order to simulate the presence of drusen in the retina, eleven different programs were built in this work. Each program has in common the basic virtual simulation scene - the eyeball surrounded by air. The difference between them is, mainly, the inclusion of new volumes, with different sizes, shapes and positions, to simulate drusen. For example, in the first program with a druse volume, Olho_S1, a spherical shape was defined by using the *G4Orb* class. The details about drusen configuration in all programs are described in the Simulation methodology section.

Each defined volume is filled with a certain material. It is thus possible to assign different characteristics to eyeball structures in an independent manner by setting a material for each structure. Considering the particles and physical processes that are applied in this work, only the material optical properties (described below) defined by the user are taken into account in simulations. The definition of the chemical composition of materials is a requirement of Geant4, although the implemented processes do not consider it in the analyzed case. The material of the world volume was defined as air and the materials of the remaining volumes were defined as pure water. Nevertheless, we stressed that all differences between eyeball structures are ensured by the different optical properties applied to each material by the user, and not by the defined chemical composition, which may be seen as indicative.

A photon beam was also included in the virtual simulation scene. This beam originates from a particle source with specific characteristics. Location, direction, size, shape, profile and particles are some of the possible properties a user can define about it.

As mentioned before, the main interest of this work was to study the interaction of low energy, or optical, photons inside the human eye. The optical photons in Geant4 are a group of photons suitable for energies below or equal to 100 eV, and the related catalogue of optical processes includes reflection and refraction at medium boundaries, bulk absorption and Rayleigh scattering [20].

Rayleigh scattering refers, after the first scientist to quantitatively study the phenomenon [24,25], to the single scattering by particles with a diameter smaller than the wavelength of the incident light.

All of the above mentioned processes were chosen to be active during the simulations in the present work. More details about the particles, processes and material properties are explained in the Optical parameters section.

### Scattering description

After the scattering process, photons travelling in an incident direction are scattered in a new direction, which may be described by the scattering coefficient, *μ*_*s*_. This coefficient is in general dependent of the incident direction of the scattered photons, and therefore the scattered intensity distribution depends on the incident direction. To describe this situation there is the scattering phase function *p*, which is defined as the normalized differential scattering coefficient.

The mean cosine of the scattering angle θ (angle between the incident and scattered directions) over the *p* distribution defines the so-called anisotropy parameter *g*. It ranges from 1 to -1, for forward and backward scattering, respectively. In isotropic scattering, it is equal to zero. In certain materials, such as biological tissues, the intensity of the scattering increases in the forward direction, and *g* tends toward the unity. The modelization of this situation is frequently done by the reduced scattering coefficient $\mu_{s}^{\prime }$, also called transport scattering coefficient, which is given by $\mu_{s}^{\prime }=\left(1-g\right)\cdot \mu_{s}$[26]. We emphasize that coefficient $\mu_{s}^{\prime }$ takes into account the anisotropy of the scattering process.

In sufficiently thick biological tissue samples, i.e. in the region of 10–100 μm [27], the interaction of scattered waves between neighboring particles cannot be ignored and multiple scattering of light becomes significant. A suitable description of this phenomenon may be accomplished, within the radiative transfer theory framework, by ignoring the wavelike behavior of light and describing the transport of photons through the absorption and scattered processes, being characterized by the absorption, *μ*_*a*_, and reduced scattering, $\mu_{s}^{\prime }$, coefficients, respectively. Under these circumstances, a stochastic approach, like the Monte Carlo implemented in Geant4, becomes appropriate to model light propagation [28].

In this work, the direction of the scattered photon was obtained following the above-mentioned stochastic approach that uses the reduced scattering coefficient ($\mu_{s}^{\prime }$) which describes the medium anisotropy together with the function (1+ cos^2^(θ)) implemented in Geant4.

### Optical parameters

The various tissues of the eyeball have different optical characteristics that were simulated in Geant4 by using optical parameters. The reflection and refraction of photons at tissue boundaries are characterized by the index of refraction, which relates to reduction of the speed of light in a medium.

We implemented the *G4OpticalPhoton* class to use optical photons in simulations and the following classes related to optical processes: *G4OpAbsorption* (for bulk absorption), *G4OpRayleigh* (for Rayleigh scattering) and *G4OpBoundaryProcess* (for reflection and refraction at medium boundaries). The optical parameters for each material used in this work are listed in Table 1[21]. RINDEX is the index of refraction used in calculations associated with the processes of reflection and refraction; ABSLENGTH and RAYLEIGH are the mean free path lengths for bulk absorption and Rayleigh scattering, respectively. All the implemented values are related to the simulated photon energy (2 eV) and were found in different publications [26,29-31]. The absorption and Rayleigh coefficients of eyeball structures are based on measured optical properties of bovine eye [26]. The optical parameters of drusen were calculated considering a composition of 50% lipids and 50% proteins, based on the work of Lan Wang et al. [32]. This composition is qualitatively similar to the milk composition presented in reference [31], which describes a study about the correlation of the absorption and reduced scattering coefficients with the fat content of milk. A highly linear relationship for both data pairs was observed and, by extrapolation, the milk coefficients were calculated considering a 50/50 composition. These coefficients were chosen to define the optical properties of drusen in this work [21].

**Table 1:** Properties of the simulated material

**Structure**	**Absorption coefficient (*μ***_**a**_**)**	**Reduced scattering coefficient (**$\mu_{s}^{\prime }$**)**	**RINDEX**	**ABSLENGTH**	**RAYLEIGH**
Air	-	-	1.000 [29]	-	-
Sclera	0.25 mm^-1^[26]	8.00 mm^-1^[26]	1.47 [30]	4.0 mm	0.1250 mm
Cornea	-	-	1.376 [29]	-	-
Aqueous humor	-	-	1.336 [29]	-	-
Iris	-	-	-	-	-
Crystalline lens	-	-	1.405 [29]	-	-
Vitreous humor	-	-	1.337 [29]	-	-
ILM	0.25 mm^-1^[26]	0.75 mm^-1^[26]	1.337^(^*^b)^	4.0 mm	1.333 mm
NR^(^*^a)^	0.25 mm^-1^[26]	0.75 mm^-1^[26]	1.337^(^*^b)^	4.0 mm	1.333 mm
RPE	90.00 mm^-1^[26]	19.20 mm^-1^[26]	1.337^(^*^b)^	0.0111 mm	0.0521 mm
Druse	12.6 cm^-1^[31]	412 cm^-1^[31]	1.337^(^*^b)^	0.7936 mm	0.0243 mm
Choroid	8.00 mm^-1^[26]	3.60 mm^-1^[26]	1.337^(^*^b)^	0.1250 mm	0.2778 mm

ABSLENGTH = 1/*μ*_a_ and RAYLEIGH = 1/$\mu_{s}^{\prime }$. (*^a^Neurosensory retina was considered to be of homogenous composition through all its thickness; *^b^Equal to vitreous humor).

It is expected the blood will not change the dispersion patterns; it will affect only the laser absorption, creating potentially shadow areas bellow the vessels. Consequently, for the sake of simplicity, in this study the blood vessels were not considered.

According to the Geant4 global definitions for optical photon processes, when a photon reaches a material without RINDEX, like the iris, it is immediately absorbed in the border of that material. When the material has no ABSLENGTH or RAYLEIGH value defined, the corresponding process doesn’t occur there. It should be mention that it wasn’t included the values of some parameters for several materials in Table 1, because the corresponding processes are not important in those materials.

### Simulation methodology

**Figure 2:**
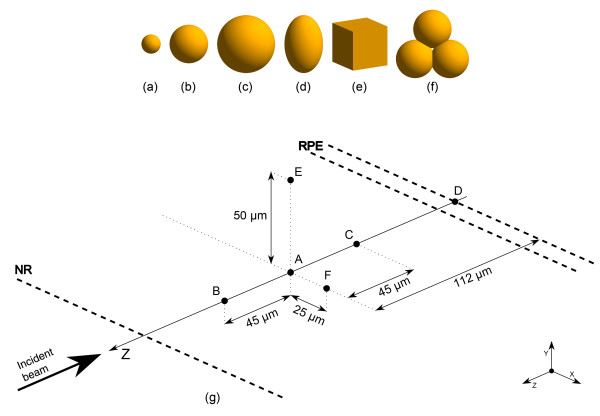
**Shapes, dimensions and positions of simulated drusen. (a)** sphere, diameter: 50 μm; **(b)** sphere, diameter: 100 μm; **(c)** sphere, diameter: 150 μm; **(d)** ellipsoid, major diameter: 150 μm, minor diameter: 100 μm; **(e)** cube, side: 100 μm; **(f)** set of spheres equal to b; **(g)** schematic diagram of drusen positions in the retina (the boundaries of NR and RPE layers are visible in the diagram).

During the simulation all developed programs ran with its drusen configuration. Figure 2 shows the shapes and dimensions of drusen built in programs.

The depth variation of drusen was also investigated in order to simulate different stages of its development, as it emerges beneath the retinal pigment epithelium (RPE) and grows towards the outer surface of the retina [33]. The various positions taken by drusen in the virtual retina are shown schematically in Figure 2(g). Each developed program combines one of the shapes with one of the positions and simulates a simple configuration of drusen in the retina. More details about all developed programs can be found in a previous reference [21].

In this work we used the OpenGL (OGL) graphics system through G4UI, the Geant4 user interface, to prepare and visualize the simulations.

Some of the properties of the particle source, as shape and position, were set by default in the program code. Others were defined at the time of simulation with the commands of the macro files. These commands control likewise the definitions of particle generation with the difference that they can be easily changed without requiring a recompilation of the program. This option has become necessary when we opted to perform simulations entailing variations in the propagation direction of the particle beam.

In all simulations, optical photons were generated with an energy of 2 eV (used for observing the fundus, it registers lower absorption in RPE layer [34]), corresponding to a wavelength of about 620 nm. The conversion between energy *E* (in eV) and wavelength λ (in nm) was done using the λ = *hc*/*E*, where *h* and *c* are the Planck’s constant and the speed of light, respectively, and *hc* =1239.842 eV nm.

**Figure 3:**
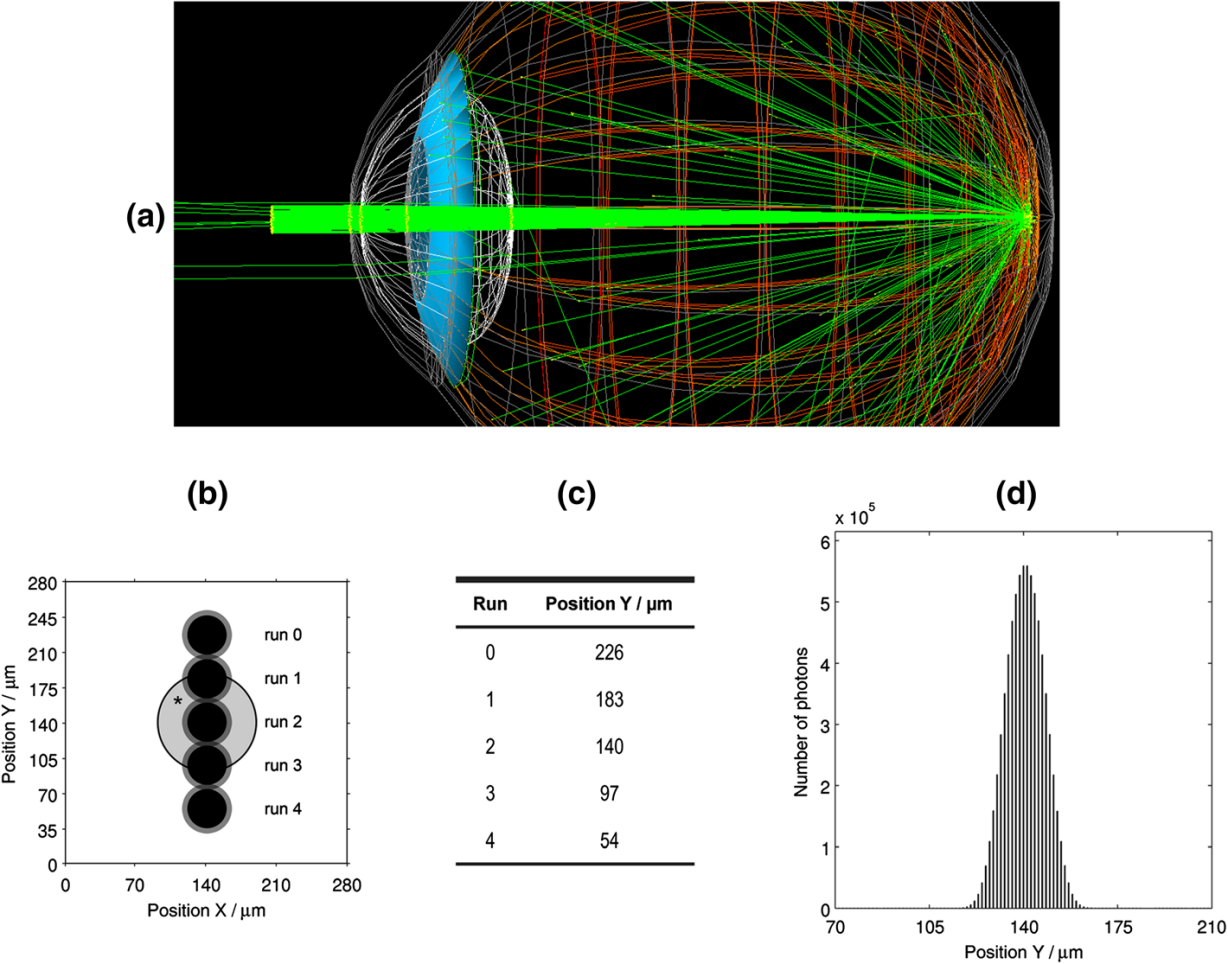
**Incidence of beam on the retina. (a)** Observation of an incident beam with 200 optical photons (green), in G4UI (OpenGL graphics); **(b)** Schematic diagram of the five incidence positions of beam on the retina (*representation of a spherical druse with a radius of 50 μm); **(c)** Position Y of the incidence points observed in a); **(d)** Histogram in Y of the beam incident on the retina, with a diameter around 50 μm (100 million simulated photons).

Besides the type and energy of particles, the format of the incident beam was also set up. A circular beam of Gaussian profile (typical laser profile) was generated outside the eye and near the cornea (Figure 3(a)), with a width at half maximum of approximately 350 μm. In the situation of the eye being accommodated to infinite, the selected laser beam dimensions will focus in the retina with a diameter of 50 μm (Rayleigh criterion). In a real situation, the focus will be dependent on the correct accommodation of the eye and the lens aberrations, being expected some loss of resolution (due to an increased beam focus diameter). In practice, increasing the laser beam width may compensate the loss of resolution. Nevertheless, this situation does not affect the result of these simulations, since the only factor that is being taken into consideration is the diameter of the laser beam focus.

In this study, were carried out vertical scans of the retina with the incident beam, similar to what is done in fundus observation techniques. For this, five runs were executed in each simulation, varying the incidence angle of the generated beam. The vector coordinates that define its rotation at the origin were selected to ensure different distances from drusen included within the retina, achieving a vertical scanning thereon.

With the aim of observing the configuration of the incident and reflected beams, inside and outside of the eye, we used virtual detectors in different locations for collecting data on the simulated photons. Each detector collected information about the number and position of photons that passed through its sensitive area. The computed information was exported to output files for analysis of the beam characteristics.

Figure 3 shows the data collected from a detector inside the eye, near the retina, where one can observe the various incidence positions of the beam on the retina (Figure 3(b)), the shape and size of the incident beam (Figure 3(d)). The number of particles of the incident beam was variable depending upon the objectives of detection (see Simulation details). Although it cannot be implemented a detector inside the eye in a real set-up, we have used it in order to better study the reflected beam.

Each detector corresponds to one layer of voxels, which count the number of optical photons that pass through a given direction. All detectors were defined with a 200 × 200 resolution. Two detectors (DA, DB) were used to register the characteristics of the incident beam, and other eight detectors (D1 to D8) were used to collect data from the reflected beam, inside and outside the eyeball. Detector DA (2.0 × 2.0 mm) was built outside the eyeball, at a distance of 1.74 mm from the cornea, and detector DB (0.28 × 0.28 mm) was built inside the eyeball, at a distance of 106 μm from the retina. Detector D1 (2.0 × 2.0 mm) was included inside the eyeball at a distance of 556 μm from retina in order to register the profile of the reflected photon beam. Outside the eyeball, several bigger detectors of the same size (8.5 × 8.5 mm) were used to collect information at different distances from cornea. These were positioned at a distance of 1 cm (D2), 2.5 cm (D3), 2.7 cm (D4), 3 cm (D5), 5 cm (D6), 7 cm (D7) or 9 cm (D8) from cornea. The data collected by all detectors were stored in text files along the simulations and used to obtain the graphs shown herein.

The photons that leave virtual eye propagate in a straight line through the air and are eliminated when they reach the frontier of the world volume.

### Simulation details

In all simulations, a total number of photons was set for the generated beam in each run. Depending on the detector location, it was necessary to generate a larger or smaller number of photons to ensure satisfactory results regarding the dispersion pattern of the beam reflected on the retina. After numerous tests, with different number of photons, it was found necessary to generate millions of photons to get well-defined beam profiles.

It was observed that the generation of a larger number of photons improves the definition of the histogram curves by decreasing the noise associated with the random, allowing a better observation of results. This increase in the photons number resulted in a proportional increase in the simulation time. In this case, it was generated a number of photons sufficient to obtain well-defined histogram curves and, at the same time, possible to be simulated on a suitable interval of time. Beams with 20 million and 200 million photons were generated for records using detectors D1 and D5, respectively.

The calculations were performed on our cluster in a Linux environment, composed of one AMD Opteron 275 at 2.2 GHz as the master node and nine Intel Core2 Quad Q6600 at 2.4 GHz as computing nodes. Each simulation ran in a single node and, for example, the time to simulate 100 million photons was about 32 hours. More details are explained in a previous reference [21].

## Results

The data collected during the simulations were processed with the help of MATLAB (version 7.12) [35]. The main results obtained for the eyeball without drusen (Olho_S0 program) and the eyeball with a spherical 100 μm druse located at an intermediate retinal depth (druse (b) centered on position A, Figure 2) (Olho_S1 program) are presented below. The overall results obtained for the other programs are also described.

### Detector inside eyeball

**Figure 4:**
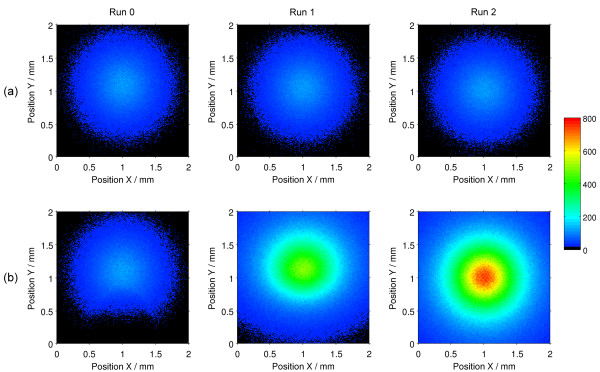
**Dispersion patterns of photons reflected in the retina, detected inside the eyeball.** Results for the first three incidence positions (Run 0 to Run 2) of the beam (direction -Z). **(a)** Results of the Olho_S0 program (without drusen in the retina); **(b)** Results of the Olho_S1 program (100 μm spherical druse in the retina). Simulation details: D1 detector; 20 million photons simulated in each run.

Figure 4 includes various graphs showing information about the photon beam that has been reflected on the retina, after its incidence on the different positions schematized in Figure 3(b). We only show the data for the Runs 0, 1 and 2, due to the symmetry of results observed in the remaining runs.

The 2D color graphs of Figure 4 show the scattering and the quantity of the photons distributed over the detector area.

In Figures 4(a) and (b) are represented the results of simulations performed with the Olho_S0 and Olho_S1 programs, respectively. The detector D1, positioned within the eyeball near the retina, collected all presented data. These graphs show the dispersion patterns of the beam reflected on the retina for each incidence position.

There are differences between the dispersion patterns of the reflected beams in both cases, and in all simulated runs. For the program that simulates the absence of drusen (Olho_S0), the results (Figure 4(a)) are very similar regarding the shape and dispersion of the reflected photon beam. In all runs we have observed a circular beam, with a central peak of greatest intensity, approximately with a Gaussian profile (more attenuated). The single variation detected over the runs boils down to the position change of the detected beam, which moves according to the change in position of the beam incident on the retina.

Analyzing the results obtained for the Olho_S1 program (Figure 4(b)), which simulates the presence of a 100 μm spherical druse in the retina, we have noticed significant differences between the various runs. In the Run 0, we have observed a beam with dispersion similar to that found in Olho_S0, attenuated in the lower central region of the detector. This attenuation occurs also in Run 4, in the upper central region of the detector.

Comparing the situation with and without drusen, illustrated in the graphs (b) and (a) (Figure 4), respectively, we have noticed that the number of photons reaching the detector in the Run 1 (b) was about 4 times higher than in the Run 1 (a), and in the Run 2 (b) was about 6 times higher than in the Run 2 (a), which indicates a larger quantity of photons reflected in the retina in the presence of the druse. The Gaussian profile of the reflected beam is similar to that of the incident generated beam, with a peak located in the central region of the detector.

**Figure 5:**
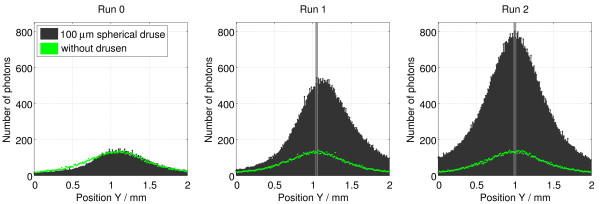
**Comparison of results obtained inside the eyeball.** Overlay of the histograms of the beams detected in the Runs 0, 1 and 2 of Olho_S0 and Olho_S1 programs. Simulation details: D1 detector; 20 million photons simulated in each run.

Histograms were also built to analyze the profile of the reflected beams, namely the maximum number of photons recorded along the position X, for each position Y of the detector. Figure 5 shows an overlay of the histograms of the beams reflected in the Runs 0, 1 and 2. In the Run 0 is visible the attenuation in the number of photons on the left side of the black graph peak (with drusen), when comparing to the green (without drusen).

In the graphs of the Runs 1 and 2 (Figure 5) a vertical grey line marks the incidence position of the beam, which is coincident with the maximum peak of the reflected beam curve in the runs related to the situation without drusen. Nevertheless, it is noticeable a deviation of the maximum peak of the curve related to the situation with drusen to this line, in the Run 1, and the coincidence of this line with the maximum peaks of the curves observed in the Run 2. Moreover, there is an asymmetry in the number of photons in the left and right sides of the black graph peak in the Run 1, which is not observed in the Run 2. This phenomenon reveals a large reflection of photons in the side of the beam incidence region and a tendency to adopt a direction perpendicular to the druse’s surface.

Considering other simulations, we have observed differences between the various reflected beams resulting from changes in size, depth, shape and position of the druse.

We have concluded that the size of the drusen influenced the profile of the beam reflected on the retina. For the smaller druse the beam proved to be less intense in the Runs 1 and 2, registering a lower number of detected photons and also a smaller curve peak. For a bigger druse a more intense beam was observed, with a higher peak and a larger quantity of photons than it was detected. In the Run 0, the attenuation observed in graphs is less visible for the smaller druse and more visible for the bigger druse.

The depth variation of the drusen also affected the profile of the reflected beam. It was found that by positioning the druse closer to the surface of the retina, the reflected beam achieved the detector with greater intensity, registering a maximum peak of photons about 17% greater than in the situation represented in Figure 5.

Changing to a deeper druse, the beam was reflected on the retina with lower intensity, with a maximum peak of photons about 12% less than the peak obtained in the curve of Olho_S1. The number of photons reaching the detector ranged around 8% by changing the depths, compared with the Olho_S1, being higher for the more superficial druse and lower for the deeper druse. Similar results were found between the simulation of the druse beneath the RPE layer (even deeper, position D, Figure 2(g)) and the simulation without the drusen (Olho_S0), with the difference that, in the first, more photons were detected in the Run 2 (about 18%) and less in the remaining (approximately 1% to 2%).

Changes in the position of the druse and in its shape influenced the reflected beam profile to the extent that the incidence of the beam over a larger surface area of the druse led to a more intense scattering of photons.

### Detector outside eyeball

With the detector D5 we have obtained the most interesting results concerning the output beam profile of the eyeball. Placed at 3 cm from the cornea, it recorded the Gaussian format of the reflected beam profiles in better detail than the other detectors, placed at longer and shorter distances. For longer distances the beams showed a more dispersed profile, while for shorter distances they were more focused on the central region of the detector.

**Figure 6:**
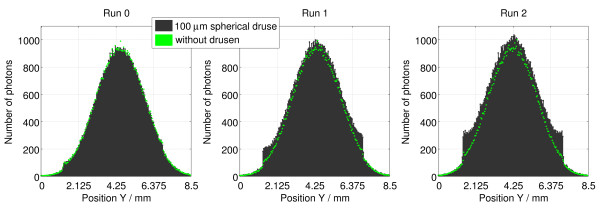
**Comparison of results obtained outside the eyeball.** Overlay of the histograms of the beams detected in the Runs 0, 1 and 2 of Olho_S0 and Olho_S1 programs. Simulation details: D5 detector; 200 million photons simulated in each run.

Figure 6 shows an overlay of these histograms, displaying the data collected by the D5 detector in comparison, similarly to what was done in Figure 5.

For the Olho_S0 program, which simulates the absence of drusen, in all runs, we have detected a Gaussian beam profile similar to the generated beam focused on the retina, the major differences between them being the levels of intensity and dispersion. The output beam has a smaller maximum intensity and a greater photon dispersion (larger beam diameter), compared to the generated incident beam (Figure 3(d)). The average percentage of photons reaching the D5 detector, from the entire generated beam, was approximately 2.8% [21,36].

From the Figure 6, we conclude that in the Run 0 photons behaved identically in both cases (with and without drusen), outside of the eyeball. Moreover, the beam attenuation that was observed by D1 detector (inside) in the Olho_S1 program (compared with Olho_S0) was not identified in the results of the D5 detector.

In the Runs 1 and 2 we noticed a greater quantity of photons, mostly in the peripheral regions of the detector, compared to Olho_S0. For the Run 1 the greatest increase occurred in the upper region. For the Run 2, the increase occurred in the whole area around the peak of the beam homogeneously.

Figure 6 shows an overlapping of the curves of the Runs 1 and 2, where we can see the differences given above from the histogram perspective. Furthermore, there was also an increase in the maximum quantities of photons that reached the pixels of the D5 detector, in both cases. The peaks maximum obtained in the Olho_S1 program (black) overlaps with the ones in the Olho_S0 program. This increasing trend is more pronounced in the Run 2.

The presence of the 100 μm spherical druse in the retina increased the number of photons in the output beam, about 0.06% in the Run 0, 15.47% in the Run 1 and 25.64% in the Run 2 (in the remaining runs there is a symmetry in the results). With the druse, the maximum percentage of photons that hit the D5 detector, from the entire generated beam, was approximately 3.5% [21].

**Figure 7:**
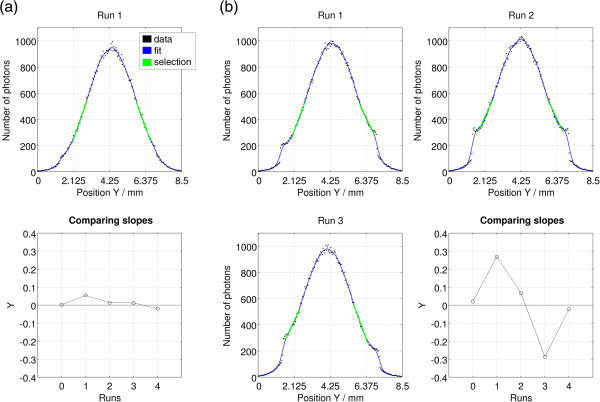
**Observation and comparison of the curve side slopes of the histograms obtained in various runs.** Each run is related to a different incidence position of the beam (see Figure 3). **(a)** Results for the Olho_S0 program; **(b)** Results for the Olho_S1 program. Simulation details: D5 detector; 200 million photons simulated in each run. Data processing details: csaps cubic smoothing spline, smoothing parameter *p* = 0.01; number of data points for linear regression: 21; height of the midpoint: 40%.

In the previous histograms asymmetries in the beam profile curves are evident and reveal the trend of dispersal of photons to the opposite side of the drusen location in relation to the beam incidence position. The side slopes of the curves are different among themselves, in absolute value, and therefore we proceeded with a comparison of them with the aim of analyzing the existent asymmetries. The resulting graphs associated with earlier results are displayed in Figure 7.

Figure 7(a) exhibits two graphs concerning the results of the situation without drusen. The top graph shows the profile curve of the output beam obtained in the Run 1, fitted with the cubic smoothing spline of MATLAB (csaps, smoothing parameter *p* = 0.01). In this graph, the regions selected for the calculation of the side slopes are represented in green. These regions were chosen considering the number of points required for the data range of each slope and the height of the chosen segment midpoint. In this case, we have chosen sets of 21 points and a height of the midpoint of 40% of the maximum height of the curve peak. The bottom graph shows the values calculated by the function *y* = *d*_1_/|*d*_2_| - 1, if *d*_1_ ≥ |*d*_2_| or *y* = *d*_2_/*d*_1_ + 1, if *d*_1_ < |*d*_2_|, for each curve identified by the respective number of the Run (*d*_*1*_ stands for the left side slope and *d*_*2*_ for the right side slope).

In the absence of the drusen (Figure 7(a)), the value calculated with *d*_*1*_ and *d*_*2*_ side slopes tends to approach the zero value in all runs, with a maximum deviation of 5% in the Run 1. The profile curve of Run 1 is one that, according to the calculations, presents less symmetry between the side slopes. The curves of the remaining runs have a shape similar to that of the Run 1, with even greater symmetry between the two sides.

In the presence of a 100 μm spherical druse (Figure 7(b)), the calculated values have a higher variability. In the Runs 0, 2 and 4 the result approaches the zero value, with deviations of 2% to 6%, whereas in the Runs 1 and 3 the deviations are, approximately, 27% and 29%, respectively. Observing the asymmetric profile curve of the output beam in the Run 1, it is evident that the left side slope (*d*_*1*_) is higher, in absolute value, than the right side slope (*d*_*2*_), thus resulting in a value greater than zero in the calculation. In the Run 3, we noticed a symmetrical profile of the Run 1, resulting in a deviation of less than zero. The curve of the Run 2 shows, visually, a greater symmetry among the side slopes, presenting a calculated value closer to those found in the runs of the simulations without the drusen. The curves of the Runs 0 and 4 have a shape similar to that in the Run 1 of Figure 7(a), presenting an identical symmetry.

Regarding the other simulations, we have noticed that the results were similar to those already described. In some cases, where spherical drusen were used, identical asymmetries were found in the beam profile curves of the Runs 1 and 3. In general, changes were related essentially to the quantity of photons and the calculated deviation values for the slopes comparison ranged between 20% and 30%. There was a lower number of reflected photons in the simulation of the 50 μm druse and in the simulation of the druse located below the RPE layer, thereby obtaining, in these cases, results closer to those observed in the simulation without the drusen.

## Discussion

The aims of this work were to simulate a photon beam interacting within an eyeball with different drusen configurations in retina and study the dispersion pattern of the reflected beam in order to evaluate the possibility of getting information about drusen’s presence.

Together, the findings indicate that the presence of drusen in the retina influences the dispersion pattern of the reflected beam, as found in another previous work that used the Geant4 [23] and a different simulation platform [37].

In general, we observed a similar behavior of photons when the beam focused totally or partially on the drusen. In these cases, it was found a greater quantity of photons reflected by the retina layers, having a tendency to assume a direction perpendicular to the surface of drusen in the way of the beam incidence region.

This phenomenon is explained by the differences in the mean free path lengths associated with the materials of retinal layers and drusen. Comparing the material of the druse with the one of the neurosensory retina (NR), which was included in most simulations, it is found that it has a mean free path length for absorption about 5 times lower (see Table 1). The scattering of photons is thus the physical process which has greater relevance within the structure of drusen (a refractive index equal to that of retinal layers was applied, canceling reflections on its surface), being higher the probability of a photon to be scattered or absorbed in the druse, comparing to the NR layer.

For example, in the Run 1 of Olho_S1 (inner detector, Figures 4(b) and 5) the beam focused partially on the druse in depth, i.e., some photons in the lower half of the photon beam have reached the structure of the druse, as other of the upper half continued its path in the NR layer. The photons that hit the structure of the druse reached a new medium with different physical conditions conferred by its characteristic material, where there is a higher probability of scattering and absorption of photons.

Thus, the result was different from that observed for the homologous situation without the druse, as a higher number of photons have been detected in the upper region of the detector. The direction changes of the photons inside the druse are more frequent than in the NR layer and, therefore, with the presence of the druse in the retina there is a higher probability to the photons take more times a path towards the detector, crossing again all the most superficial retinal layers and the vitreous humor to achieve it. This fact also explains the attenuation observed in some reflected beams when the incidence occurred in a peripheral region of the druse (e.g., Run 0, Olho_S1, Figure 4(b)). Photons that were scattered in the NR layer may have reached the druse, after a few deviations, and be further dispersed in other directions different from that towards the detector, reducing the detected beam intensity in the region related to the position of the druse in the retina.

When the beam was focused entirely on the druse central region, we obtained a larger number of reflected photons, since in these cases the majority of the beam photons focused on the druse and traveled through its inner volume, thereby existing higher probability of reflection. In these cases (e.g., Run 2, Figure 4(b)) there was greater symmetry in the dispersion pattern of the reflected beams, since the beam incidence was performed symmetrically on the druse front surface. This symmetry is indicative of the tendency of the photons in assuming a direction perpendicular to the surface of drusen. In other situations (e.g., Run 1, Figure 4(b)) the beam incidence occurred laterally, in an asymmetrical way, giving rise to asymmetric dispersion patterns in the reflected beam.

It was also verified that the drusen geometry influences the results. The variation in size, shape or position led to different surface areas and volumes of drusen to be exposed to different numbers of incident photons in several simulated runs. The scattering of photons occurred preferentially in the direction perpendicular to the surface of incidence in drusen, opposite to the location of the higher volume of its dispersive material. Therefore, drusen with larger volumes or wider shapes originated more intense reflections at the time of incidence of the photon beam, due to the greater probability of interaction.

Deeper drusen likewise interacted with the incident beam. However, the beam hit them with less intensity since the probability of interaction with the material of the retinal layers was greater due to the increased distance traveled by the photons. On the other hand, the photons scattered in the druse traveled back through the same depth to reach the detector, doubling the interaction with the retinal layers. Thus, more photons were deflected or absorbed in the retina, causing the beam to become less intense for deeper drusen. In the situation in which we simulated the druse beneath the RPE layer, even deeper, we observed a set of results similar to those found in the simulation without drusen. This emphasizes the absorbing nature of the RPE layer, which has the highest absorption coefficient of all materials used in the programs. Most of the photons that reached the depth of this layer were absorbed and not scattered towards the detector.

Changes in the reflected beam patterns were observed inside and outside the eyeball, with less intensity outside. The differences are due to the occurrence of absorption of photons from the beam periphery. Many photons diverged and propagated towards the walls and the anterior internal structures of the eyeball, failing to reach the outside detector and to contribute to the shape of the output beam profile. Despite the differences, this result indicates that, under the experienced conditions, it may be possible to detect characteristics of the output beam that can provide information about the presence of drusen in the retina.

The main difference between the results obtained outside of the eyeball lies in the number of photons detected in the peripheral regions of the beams reflected at various runs. This is related with the observations inside the eyeball, being justified by the interaction between the photons and the dispersive material of drusen, as explained before. Note that, only the central part of the reflected beam reached the D5 detector, outside, due to its divergence and absorption inside, and therefore less noticeable differences were detected in the beam profile, compared to those observed within the eyeball. To simplify the observation of the found differences we proceeded to a comparison of the side slopes of the output beam profile curves. This comparison has shown the existence of asymmetries in the profile curves of the beams detected in some runs of the simulations with drusen.

The analysis of the reflected beams with asymmetric profile curves enabled the collection of information about drusen. Depending on the size, shape and position of drusen, the asymmetries of the beams reflected in the scanning have been more or less intense, being related to the distribution of photons in the peripheral region of the beams. The largest calculated deviations were identified in the Runs 1 and 3, which are related to a lateral incidence of the beam on the druse. This lateral incidence led to an increased quantity of detected photons in one of the sides of the detector, originating asymmetries in the profile curve of the reflected beam. For example, the fact that the deviation calculated for the Run 1 of the Olho_S1 program (Figure 7(b)) is greater than zero indicates that the right side slope (*d*_*2*_) is lower (in absolute value) than the left side slope (*d*_*1*_), meaning that there is a greater quantity of photons on the right side of the beam profile curve, i.e., more photons were detected in the upper region of the detector. In this case, the larger the deviation in the graph comparative of the slopes, the greater the quantity of photons in one side of the detector. In cases of frontal incidence or absence of drusen this asymmetry was practically not observed and the calculated deviations (below 10%) may be considered as uncertainty values associated with process statistics.

In these conditions, the asymmetries of the beam profile curve thus constitute an indicator of the existence of an interaction between the incident beam and a border region of the druse. The analysis of the number of detected photons was also important to this process, as it allowed the collection of more information about the configuration of the identified druse, by comparison. Depending on, for example, its volume or its depth, different quantities of photons were reflected, influencing the shape of the beam profile curves.

Thus, it was observed the possibility to identify outside of the eyeball the characteristics of the reflected beam that can reveal the presence of drusen in the retina, located beneath the RPE layer. The intensity of these characteristics, although lower to that found within, proved to be sufficient for the possibility of detection.

In this work we assigned the value of the refractive index of the vitreous humor to all retinal layers and to the structure of drusen, with the aim of analyzing only the effects of the Rayleigh scattering and the photon absorption therein, as in the work of Branco [37]. It is considered that the processes of reflection/refraction at the contact surfaces between the layers only slightly influence the intensity of results, taking into consideration the proximity of the values of the refractive indices of biological tissues. The genetic variability associated with tissues is a factor to take into account, since it may influence the quality of the results.

In the future it may be interesting to run new simulations with programs that increase the complexity of the eyeball, making the results even closer to the experimental reality. For this, it is advisable to collect even more detailed information about the parameters associated with the physiological components of the eye.

The study of new methods for interpreting the dispersion patterns of reflected beams may also be a relevant investigation to the development of future diagnostic processes, as drusen detection, and the validation of the SLO retro-mode.

## Conclusions

The simulations performed with Geant4 revealed some new insights about the way a laser beam interacts with drusen during retinal scanning techniques. The results indicate that the presence of drusen in the retina influences the dispersion pattern of the reflected beam, even in deep locations near the RPE layer. The analysis of the output beam profile curves in simulations with drusen revealed the existence of asymmetries, which constitute an indicator of drusen’s presence. The shape of beam profile curves depends on the characteristics of the druse and its location relative to laser incidence position.

In conclusion, it was observed that, under the experienced conditions, it might be possible to detect characteristics of the output beam that can provide information about the presence of drusen in the retina. The analysis of beams reflected in retinal laser scanning techniques could be included as a diagnostic examination hypothesis to prevention of AMD, contributing to maintain the visual health of the population.

## Abbreviations

AMD: Age-related macular degeneration; EM: Electromagnetic; G4UI: Geant4 user interface; ILM: Internal limiting membrane; MC: Monte Carlo; NR: Neurosensory retina; OCT: Optical coherence tomography; OGL: OpenGL; RPE: Retinal pigment epithelium; SLO: Scanning laser ophthalmoscopy.

## Competing interests

The authors declare that they have no competing interests.

## Authors’ contributions

DT conceived and designed the study, computed the simulations, collected, analyzed and interpreted the data, built tools for data visualization and analysis, wrote and organized the manuscript, built the figures; GL contributed to conception and design of the study, acquisition and analysis of data; PV proposed the idea of this study, participated in its design and coordination, analyzed and interpreted the data, took part in writing Abstract and Background, collaborated in the review of the manuscript. JPS collaborated with the idea of this study, oriented this work, analyzed and interpreted the data, collaborated in writing and in the review of the manuscript. All authors read and approved the final manuscript.

## References

[B1] Tanaka Y Shimada N Ohno-Matsui K Hayashi W Hayashi K Moriyama M Yoshida T Tokoro T Mochizuki M Retromode retinal imaging of macular retinoschisis in highly myopic eyes Am J Ophthalmol 2010 149 635 640 e631 10.1016/j.ajo.2009.10.024 20346779

[B2] Acton JH Cubbidge RP King H Galsworthy P Gibson JM Drusen detection in retro-mode imaging by a scanning laser ophthalmoscope Acta Ophthalmol 2011 89 e404 e411 10.1111/j.1755-3768.2011.02123.x 21332676

[B3] Kinnunen K Petrovski G Moe MC Berta A Kaarniranta K Molecular mechanisms of retinal pigment epithelium damage and development of age-related macular degeneration Acta Ophthalmol (Copenh) 2012 90 299 309 10.1111/j.1755-3768.2011.02179.x 22112056

[B4] Preece SJ Claridge E Monte Carlo modelling of the spectral reflectance of the human eye Phys Med Biol 2002 47 2863 2877 10.1088/0031-9155/47/16/303 12222851

[B5] Nelson WR Hirayama H Rogers DWO Report SLAC-26 1985

[B6] Ferrari A Sala PR Fasso A Ranft J Report SLAC-R-773 2005

[B7] Briesmeister JF Los Alamos National Laboratory Report LA-13709-M 2000

[B8] Agostinelli S Allison J Amako K Apostolakis J Araujo H Arce P Asai M Axen D Banerjee S Barrand G Behner F Bellagamba L Boudreau J Broglia L Brunengo A Burkhardt H Chauvie S Chuma J Chytracek R Cooperman G Cosmo G Degtyarenko P Dell'Acqua A Depaola G Dietrich D Enami R Feliciello A Ferguson C Fesefeldt H Folger G Geant4 - a simulation toolkit Nucl Instrum Methods Phys Res A 2003 506 250 303 10.1016/S0168-9002(03)01368-8

[B9] Allison J Amako K Apostolakis J Araujo H Dubois PA Asai M Barrand G Capra R Chauvie S Chytracek R Cirrone GAP Cooperman G Cosmo G Cuttone G Daquino GG Donszelmann M Dressel M Folger G Foppiano F Generowicz J Grichine V Guatelli S Gumplinger P Heikkinen A Hrivnacova I Howard A Incerti S Ivanchenko V Johnson T Jones F Geant4 developments and applications IEEE Trans Nucl Sci 2006 53 270 278

[B10] Santina G Nieminen P Evansa H Daly E Lei F Truscott PR Dyer CS Quaghebeur B Heynderickx D New Geant4 based simulation tools for space radiation shielding and effects analysis Nucl Phys B (Proc Suppl) 2003 125 69 74

[B11] Geant4-DNA project [http://geant4-dna.org]

[B12] Boas DA Culver JP Stott JJ Dunn AK Three dimensional Monte Carlo code for photon migration through complex heterogeneous media including the adult human head Opt Express 2002 10 159 170 10.1364/OE.10.000159 19424345

[B13] Fang Q Mesh-based Monte Carlo method using fast ray-tracing in Plucker coordinates Biomed Opt Express 2010 1 165 175 10.1364/BOE.1.000165 21170299 PMC3003331

[B14] Fang Q Boas DA Monte Carlo simulation of photon migration in 3D turbid media accelerated by graphics processing units Opt Express 2009 17 20178 20190 10.1364/OE.17.020178 19997242 PMC2863034

[B15] Wang L Jacques SL Zheng L MCML—Monte Carlo modeling of light transport in multi-layered tissues Comput Methods Programs Biomed 1995 47 131 146 10.1016/0169-2607(95)01640-F 7587160

[B16] Geant4 [http://geant4.web.cern.ch/geant4]

[B17] Carrier JF Archambault L Beaulieu L Roy R Validation of GEANT4, an object-oriented Monte Carlo toolkit, for simulations in medical physics Med Phys 2004 31 484 492 10.1118/1.1644532 15070244

[B18] Amako K Guatelli S Ivanchencko V Maire M Mascialino B Murakami K Pandola L Parlati S Pia MG Piergentili M Sasaki T Urban L Geant4 and its validation Nucl Phys B Proc Suppl 2006 150 44 49

[B19] Glaser AK Kanick SC Zhang R Arce P Pogue BW A GAMOS plug-in for GEANT4 based Monte Carlo simulation of radiation-induced light transport in biological media Biomed Opt Express 2013 4 741 759 10.1364/BOE.4.000741 23667790 PMC3646601

[B20] Geant4, physics reference manual [http://geant4.web.cern.ch/geant4/UserDocumentation/UsersGuides/PhysicsReferenceManual/fo/PhysicsReferenceManual.pdf]

[B21] Tendeiro D Implementação de um sistema para simulação por Monte Carlo da passagem de fotões através do olho humano mediante a utilização da plataforma GEANT4 - I. Master’s Thesis 2012 Universidade Nova de Lisboa [http://hdl.handle.net/10362/7704]

[B22] Kasthurirangan S Markwell EL Atchison DA Pope JM MRI study of the changes in crystalline lens shape with accommodation and aging in humans J Vis 2011 11 1 16 10.1167/11.3.19 21441300

[B23] Lopes G Implementação de um Sistema Para Simulação por Monte Carlo da Passagem de Fotões Através do Olho Humano Mediante a Utilização da Plataforma GEANT4 - II. Master’s Thesis 2012 Universidade Nova de Lisboa [http://hdl.handle.net/10362/7705]

[B24] Strutt JW On the light from the sky, its polarisation and colour Philos Mag 1871 41 107 120

[B25] Strutt JW On the light from the sky, its polarisation and colour Philos Mag 1871 41 274 279

[B26] Rovati L Cattini S Viola F Staurenghi G Measuring dynamics of scattering centers in the ocular fundus Int J Smart Sens Intell Syst 2008 1 799 811

[B27] Flock ST Wilson BC Patterson MS Total attenuation coefficients and scattering phase functions of tissues and phantom materials at 633 nm Med Phys 1987 14 835 841 10.1118/1.596010 3683313

[B28] Firbank M Arridge SR Schweiger M Delpy DT An investigation of light transport through scattering bodies with non-scattering regions Phys Med Biol 1996 41 767 783 10.1088/0031-9155/41/4/012 8730669

[B29] Hecht E Optics 2002 4 San Francisco: Addison Wesley

[B30] Bashkatov AN Genina EA Kochubey VI Tuchin VV Estimation of Wavelength Dependence of Refractive Index of Collagen Fibers of Scleral Tissue Proc SPIE 4162, Controlling Tissue Optical Properties: Applications in Clinical Study 2000 265 268

[B31] Qin J Lu R Measurement of the absorption and scattering properties of turbid liquid foods using hyperspectral imaging Appl Spectrosc 2007 61 388 396 10.1366/000370207780466190 17456257

[B32] Wang L Clark ME Crossman DK Kojima K Messinger JD Mobley JA Curcio CA Abundant lipid and protein components of drusen PLoS One 2010 5 e10329 10.1371/journal.pone.0010329 20428236 PMC2859054

[B33] Hageman GS Luthert PJ Victor Chong NH Johnson LV Anderson DH Mullins RF An integrated hypothesis that considers drusen as biomarkers of immune-mediated processes at the RPE-Bruch’s membrane interface in aging and age-related macular degeneration Progr Retin Eye Res 2001 20 705 732 10.1016/S1350-9462(01)00010-6 11587915

[B34] Spaide RF Curcio CA Drusen characterization with multimodal imaging Retina 2010 30 1441 1454 10.1097/IAE.0b013e3181ee5ce8 20924263 PMC2952278

[B35] MATLAB [http://www.mathworks.com/products/matlab]

[B36] Van Norren D Tiemeijer LF Spectral reflectance of the human eye Vis Res 1986 26 313 320 10.1016/0042-6989(86)90028-3 3716223

[B37] Branco L Implementação de um Sistema de Simulação por Monte Carlo de Fotões Através do Olho Humano. Master’s Thesis 2009 Universidade Nova de Lisboa [http://hdl.handle.net/10362/3930]

